# MZmine 2: Modular framework for processing, visualizing, and analyzing mass spectrometry-based molecular profile data

**DOI:** 10.1186/1471-2105-11-395

**Published:** 2010-07-23

**Authors:** Tomáš Pluskal, Sandra Castillo, Alejandro Villar-Briones, Matej Orešič

**Affiliations:** 1 G0 Cell Unit, Okinawa Institute of Science and Technology (OIST), Onna, Okinawa, Japan; 2 Quantitative Biology and Bioinformatics, VTT Technical Research Centre of Finland, Espoo, Finland

## Abstract

**Background:**

Mass spectrometry (MS) coupled with online separation methods is commonly applied for differential and quantitative profiling of biological samples in metabolomic as well as proteomic research. Such approaches are used for systems biology, functional genomics, and biomarker discovery, among others. An ongoing challenge of these molecular profiling approaches, however, is the development of better data processing methods. Here we introduce a new generation of a popular open-source data processing toolbox, MZmine 2.

**Results:**

A key concept of the MZmine 2 software design is the strict separation of core functionality and data processing modules, with emphasis on easy usability and support for high-resolution spectra processing. Data processing modules take advantage of embedded visualization tools, allowing for immediate previews of parameter settings. Newly introduced functionality includes the identification of peaks using online databases, MS^n ^data support, improved isotope pattern support, scatter plot visualization, and a new method for peak list alignment based on the random sample consensus (RANSAC) algorithm. The performance of the RANSAC alignment was evaluated using synthetic datasets as well as actual experimental data, and the results were compared to those obtained using other alignment algorithms.

**Conclusions:**

MZmine 2 is freely available under a GNU GPL license and can be obtained from the project website at: http://mzmine.sourceforge.net/. The current version of MZmine 2 is suitable for processing large batches of data and has been applied to both targeted and non-targeted metabolomic analyses.

## Background

Mass spectrometry (MS) coupled with online separation methods, such as liquid chromatography (LC), is commonly applied for differential and quantitative profiling of biological samples in metabolomic and proteomic research. Such approaches are useful in the domains of systems biology, functional genomics, and biomarker discovery. One of the ongoing challenges of such molecular profiling approaches is the development of better data processing methods. Several software packages have been developed for this purpose, and have been extensively reviewed by Katajamaa and Orešič [1].

The recent introduction of mzML, an open and universal format for MS data [2], represents an important milestone in the effort to address the issues of MS data exchange and standardization. It also underlines the need for a flexible and universal software framework to provide the necessary support for data import, export, and visualization, thus allowing the rapid development of specialized data-processing methods.

MZmine was first introduced in 2005 as an open-source software toolbox for LC-MS data processing [3]. The first version of MZmine defined the data analysis workflow and implemented simple methods for data processing and visualization [3,4]. The software has been applied to numerous metabolomic analyses [5-10] and comparative studies with other related software packages have been performed [9,11]. A weakness of MZmine was insufficient modularity in its initial design, thus limiting the possibility of expanding the software with new methods developed by the scientific community. For this reason, the new release, MZmine 2, was completely redesigned to support modularity. Here we describe the architecture of MZmine 2 as well as its basic features. We also introduce a new and efficient method for peak list alignment that was implemented in MZmine 2.

## Implementation

MZmine 2 was developed using Java technology, and is therefore platform independent. The software has been tested on the Windows, Mac OS X, and Linux platforms. We focused on three main aims during the software design and implementation.

**Figure 1:**
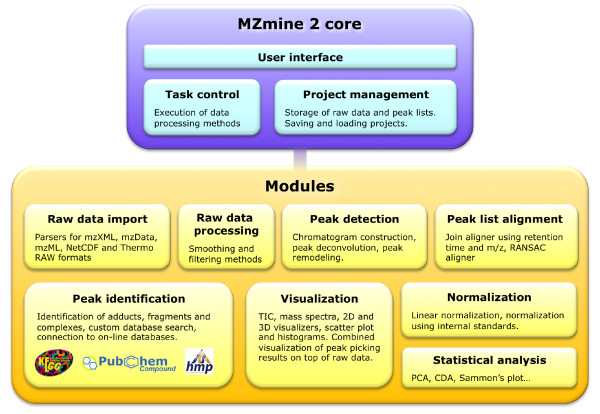
MZmine 2 software architecture and its main modules

First, the framework should be flexible and allow for easy and straightforward development of new data processing modules. We addressed this by keeping a strict separation between the application core and individual modules for data processing and visualization (Figure 1). A compact data model was designed and the code of each Java class code was kept short and intuitive. To support the development of new modules, we provided an online tutorial available at the project web site.

Second, the graphical interface of the application should be intuitive and easy to use. For this purpose, critical data processing methods such as peak picking were linked to embedded visualization modules, providing online previews during parameter setup. Additionally, the use of any data-processing method in MZmine 2 does not remove the original (unprocessed) data, giving the user the option to return back to previous results or raw data at any stage of data processing.

The third goal was to provide good support for processing high-resolution MS data, *e.g.*, as obtained from Orbitrap or Fourier transform ion cyclotron resonance MS instruments. We designed the data import and peak detection modules to maintain the precision of the imported data without any degradation due to inadequate resampling. Because the use of high-resolution data suggests an increased data volume, MZmine 2 was tested and optimized with large datasets (on the order of gigabytes).

The flexibility of the Java environment allows MZmine 2 to take advantage of several open-source libraries, including JFreeChart (http://www.jfree.org/jfreechart/) for the TIC, spectra, 2D and other visualizers, VisAD (http://www.ssec.wisc.edu/~billh/visad.html) for the 3D visualizer, Chemistry Development Kit (CDK) [12] for calculating isotopic distributions, JChemPaint (http://jchempaint.sourceforge.net/) for rendering 2D molecular structures, and Jmol (http://jmol.sourceforge.net/) for rendering 3D molecular structures. These libraries are included in the MZmine 2 distribution.

## Results

The typical MS data processing workflow comprises raw data file import, filtering/smoothing (optional), peak picking, peak list deisotoping, alignment, gap filling, and normalization [4]. The MZmine 2 modules cover all these workflow stages and also include additional functionality for the visualization and interpretation of the results. Only features new to MZmine 2 are described in this section.

### Project management

One of the new core features of MZmine 2 is project management, which allows the user to track and store intermediate results. Each data-processing step can be performed multiple times with different parameters and the results can be observed and compared. The data processing pipeline settings (e.g., algorithms and parameters used, reference peak lists) can be stored for future applications. Direct export of the peak list data to comma-separated values (CSV) or XML files is also possible.

### Raw data file format support

MZmine 2 can read and process both unit mass resolution and accurate mass resolution MS data in both continuous and centroid modes, including fragmentation (MS^n^) scans. Raw data import is modularized and the currently supported file formats are mzML (1.0 and 1.1), mzXML (2.0, 2.1 and 3.0), mzData (1.04 and 1.05), NetCDF, and RAW format used natively by Thermo Fisher Scientific instruments (requires installation of Thermo Xcalibur). Support for other file formats can be implemented as additional plug-ins.

### Data visualization

**Figure 2:**
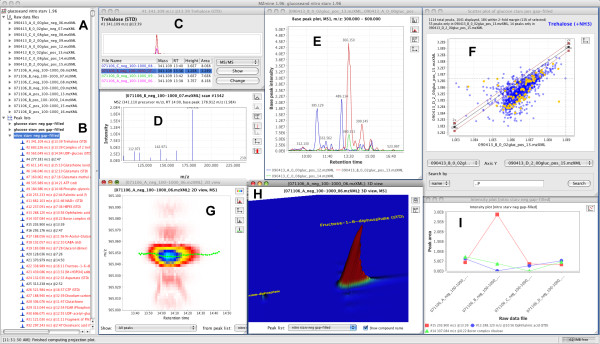
**Screenshot of MZmine 2 showing multiple visualization modules**. The specific panels included are: (A) imported samples, (B) peak lists including single peak list contents, (C) peak shapes for an identified metabolite across multiple samples, (D) MS/MS spectrum of a metabolite, (E) combined base peak plot for multiple samples, (F) scatter plot of peak areas across two samples, (G) 2D plot of a detected peak, mass-to-charge ratio vs. retention time, (H) 3D view of a detected peak, and (I) intensity plot for specific peaks across multiple samples.

MZmine 2 includes several of visualization modules (Figure 2), all of which were newly implemented for this release. Following the goal of providing the user with an intuitive interface, the visualizers automatically annotate raw data with the obtained peak picking and identification results, allowing for quick orientation when large amounts of data are being processed.

Quantitative results in the form of peak lists may be observed using a table visualizer or chart-plotting modules (Figure 2I). The scatter plot visualizer (Figure 2F) has proven to be very useful for efficient comparison of multiple samples [13].

### Peak detection

**Figure 3:**
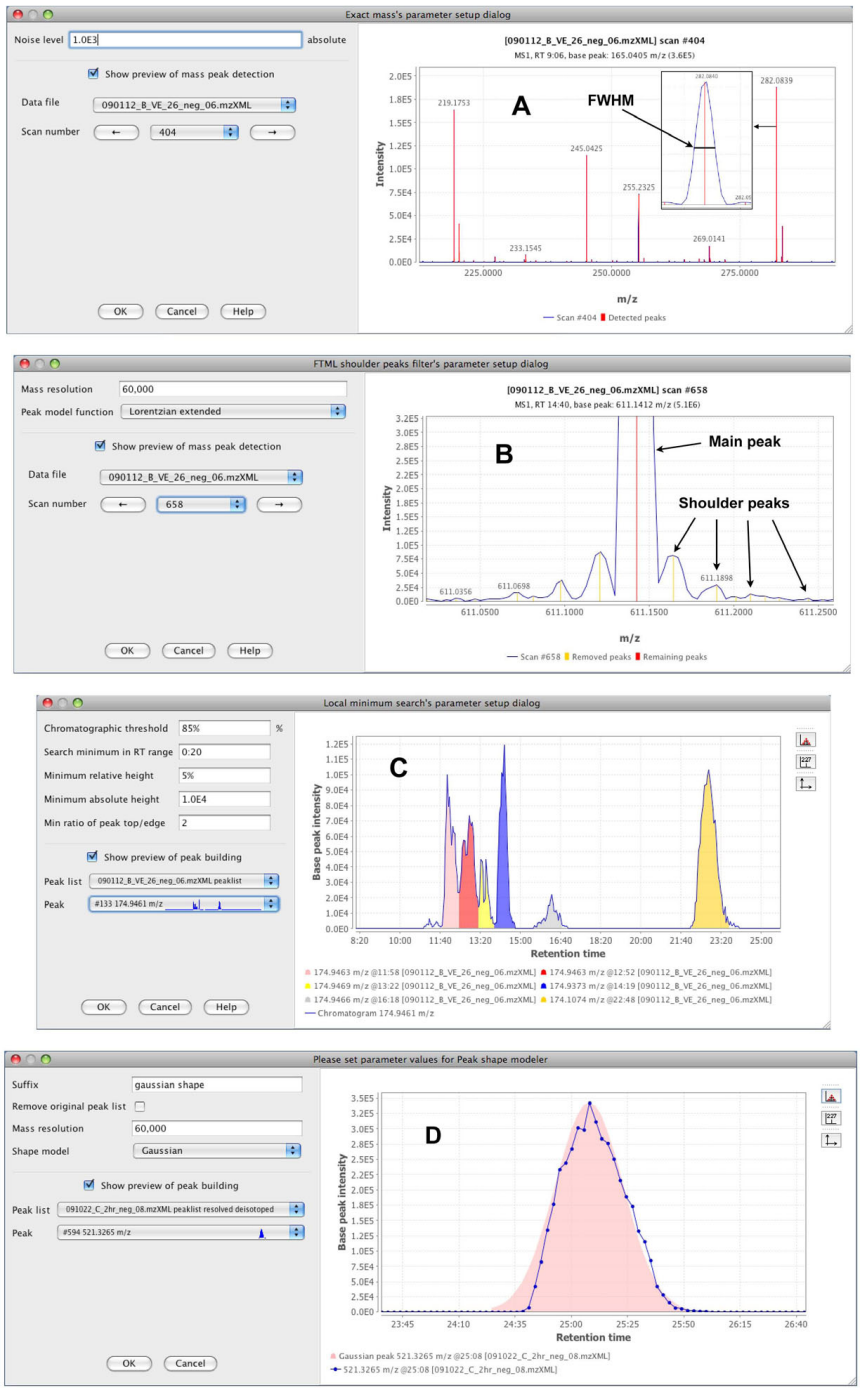
**Peak detection modules with previews**. (A) Mass detection (centroiding) module. Recognized m/z peaks are shown in red. In the insets, details of a single m/z peak are shown, indicating the full width at half maximum approach to the m/z value calculation. (B) Fourier transform mass spectrometry shoulder peaks filter. In the preview panel, the main detected peak is indicated with the red line, while shoulder peaks are indicated with the yellow lines. (C) Peak deconvolution. Each individual recognized peak within the chromatogram is indicated by a different color. (D) Experimental peak shape modeler. A Gaussian peak model (pink) is fitted to the deconvoluted chromatographic peak's data points (blue).

Feature detection is a critical step in MS data processing. The peak detection methods and their implementations should be flexible enough to deal with great differences in data obtained from different instruments, such as variable mass resolution, chromatographic resolution and peak shape, or background noise. In MZmine 2, peak detection is performed in several customizable steps (Figure 3). Previews are provided to allow for optimal selection of parameter values.

In the first step (Figure 3A), each MS spectrum is processed individually and converted to pairs of m/z and intensity values (in other words, each mass spectrum is centroided). Several algorithms are provided as plug-ins, each suitable for a different type of mass spectra. The "Local maxima" algorithm is a simple algorithm suitable for demonstrating the process: it detects each local maximum in the spectrum. The "Recursive threshold" algorithm is based on an earlier method implemented in MZmine [3,4] and adds two additional parameters of minimum and maximum peak m/z width. This method reduces the false positives by avoiding detection of noise peaks. The "Wavelet transform" algorithm is particularly suitable for noisy data. It processes each spectrum using continuous wavelet transform, matching the m/z peaks to the "Mexican hat" wavelet model. This algorithm is based on a previously reported method [14]. The "Exact mass" algorithm assumes high quality spectra (high mass resolution, low noise) and determines the center of each m/z peak using the "full width at half maximum" paradigm: m/z value is placed in the middle of the line, which crosses the peak at half of the maximum intensity (as shown in the insets in Figure 3A). Finally, the "Centroid" algorithm is suitable for already centroided data. It detects all data points above the specified noise level as m/z peaks.

Data obtained by Fourier transform mass spectrometry instruments provide very high mass resolution, but suffer from the presence of noise signals known as "shoulder peaks" (Figure 3B). These peaks are residues of the Fourier transform function calculated by the instrument and their intensity is usually below 5% of the intensity of the main (true) m/z peak. To remove these noise peaks, we introduced an optional filtration plug-in that builds a theoretical model (such as Gaussian or Lorentzian) with given mass resolution around each peak, and removes all noise peaks below this model. Peaks are processed in the order of decreasing intensity. In the preview (Figure 3B), the main m/z signal is indicated by the red color, while the shoulder peaks subject to removal are indicated in yellow. Again, it is possible to implement other filtration algorithms as plug-ins.

The next step consists of an algorithm that connects consecutive m/z values spanning over multiple scans into chromatogram objects. The default algorithm provided by MZmine 2 connects m/z values in the order of their intensity, with the most intense peaks connected first. A chromatogram spanning a given minimal time range is constructed for each m/z value (within user-defined tolerance). Each chromatogram is then deconvoluted into individual chromatographic peaks (Figure 3C). Several algorithms are provided as plug-ins. The "Baseline cut-off" algorithm recognizes each chromatographic peak that has an intensity above a given minimum level and spans over a given minimum time range. The "Noise amplitude" algorithm adds another parameter specifying the intensity range, which is considered noisy. The algorithm then finds the intensity level where most of the noise is concentrated and sets the baseline level to this intensity, individually for each chromatogram. Following the setting of the baseline, the procedure is the same as the "Baseline cut-off" algorithm. The Savitzky-Golay algorithm uses the smoothed second derivative of the chromatogram curve to detect the borders of individual peaks. The "Local minimum search" algorithm attempts to identify local minima in the chromatogram as border points between individual peaks. Several restrictions are placed on possible peak shapes, such as minimum absolute and relative intensities, or a minimum ratio between peak maximum and edge.

We also implemented an experimental module, which fits the (potentially noisy) set of data points of each deconvoluted peak with an ideal peak model such as Gaussian or Exponentially Modified Gaussian (Figure 3D). Such an approach may reduce the chromatographic noise between samples, but the practical applicability of this method has not yet been thoroughly validated.

### Peak identification

**Figure 4:**
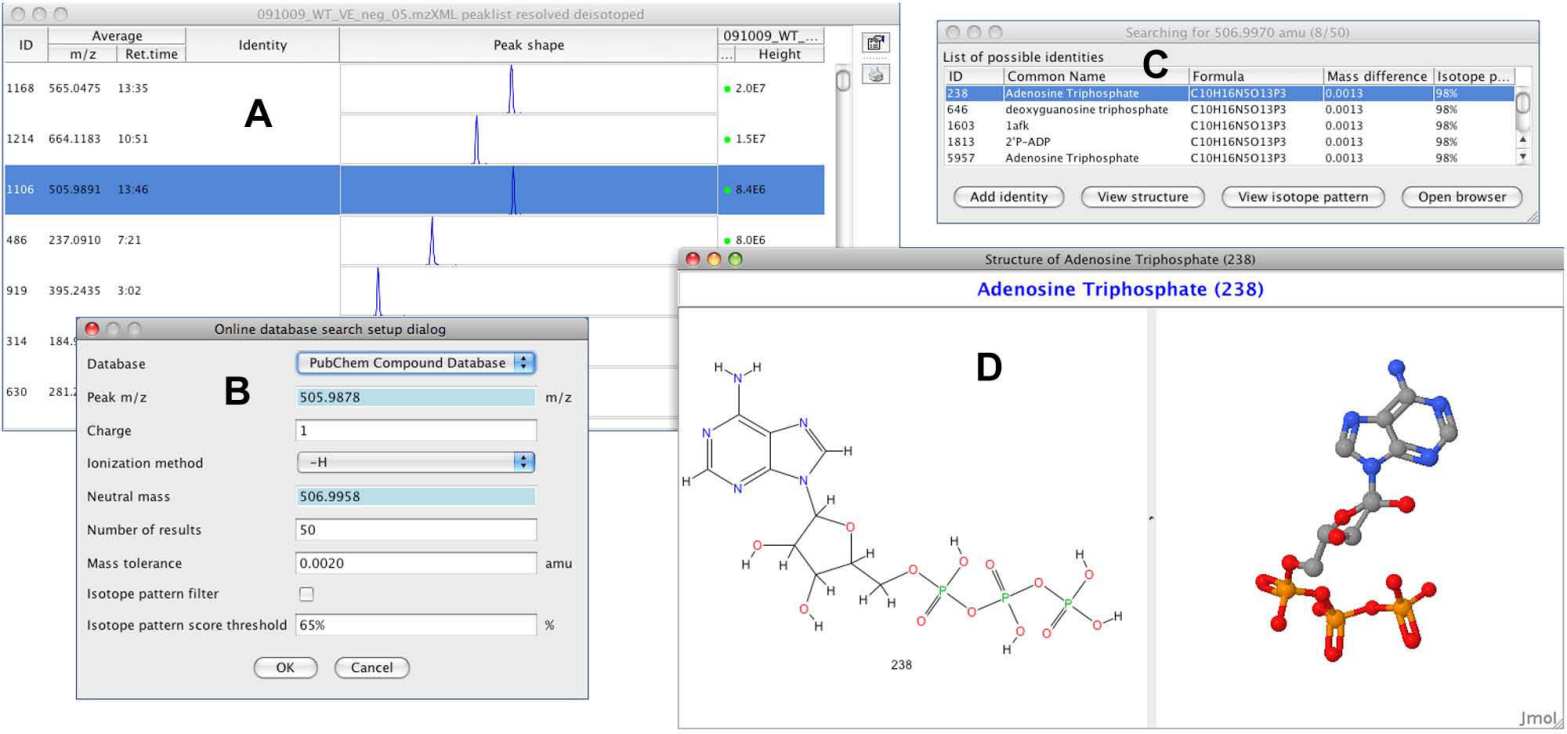
**Peak identification using the PubChem Compound database**. (A) A peak list showing the row selected for identification. (B) Dialog for setting search parameters. (C) Table of candidates obtained from the database within a given mass tolerance. (D) 2D and 3D structural views of the candidate compound.

Assignment of intuitive metabolite or peptide names to detected m/z values greatly assists with the process of data interpretation. In MZmine 2, identification of peaks can be performed either by searching a custom database of m/z values and retention times, or by connecting to an online resource such as PubChem [15], KEGG [16], METLIN [17], or HMDB [18] directly from the MZmine 2 interface (Figure 4). For each ion subjected to identification, its neutral molecular mass (*m*_*neutral*_) is calculated from its m/z value. For that purpose, the charge of the ion (*z*) can be automatically determined from its isotope pattern. Ionization mode (positive or negative) and ionization adduct (*e.g. *H^+^, Na^+^, K^+^, etc.) are selected by the user as parameters. Neutral mass is then calculated as *m*_*neutral *_= (m/z × *z*) ± *m*_adduct_, where the sign (±) is defined by the ionization mode and *m*_adduct _is the mass of the selected ionization adduct. The neutral mass *m*_*neutral *_is the primary term for database search, within user-specified tolerance. Isotopic pattern similarity can be used as a second filter to select optimal candidates, by comparing the ratios of the detected isotopes and matching isotopes from the predicted isotopic pattern of the database compound. Because the online identification module is itself modularized, support for other molecular databases can be easily added. For proteomic applications, a module allowing identification of peptide peaks using the MASCOT [19] search engine and MS/MS spectra is under development.

### RANdom SAmple Consensus (RANSAC) aligner

The purpose of peak list alignment is to match relevant peaks across multiple samples. The original MZmine software introduced a simple alignment algorithm that first creates an empty master peak list and then aligns each peak from given peak lists (samples) to the best candidate of the master list using a two-dimensional alignment window (AW) represented by user-specified m/z and retention time tolerances. If no suitable candidate is found, a new row is created in the master list. In MZmine 2, this algorithm is referred to as the "Join aligner". One disadvantage of the Join aligner is the inability to cope with a non-linear deviation of the retention times among samples. For this purpose, we introduced a new peak list alignment method based on the RANSAC algorithm.

The RANSAC algorithm [20] is a non-deterministic iterative algorithm that estimates parameters of a mathematical model from a set of observed data, which may include outliers. The probability of obtaining a good result increases with the number of iterations. In each iteration, a random subset of observed data points is selected and a model is fit to this data. In our specific case, we used 4 points to find a non-linear model. The remaining data is tested against the fitted model and if a value fits well, it is considered a part of the model. Finally, the model is evaluated and when the iteration is finished, the model with the most data points fitted to it is considered the best.

The RANSAC method of alignment makes use of two user-defined two-dimensional windows, the RANSAC window (RW) and Alignment window (AW), respectively. The RW is defined by the m/z threshold rm_0 _and retention time threshold rr_0_, and AW constitutes the same m/z threshold rm_0 _but a different retention time threshold ar_0_. The retention time threshold in RW should be as big as the maximum observed deviation in the retention time among all peaks. The procedure for aligning a sample *S*_*j *_with the master list *L *is as follows:

**Step 1**: For every row *i *in *L*, let

*r*_*i *_= the average retention time of all individual peaks in the row

*m*_*i *_= average m/z of all individual peaks in the row

*RW*_*i *_= [(*m, r*) | *m*_*i *_- *rm*_*0 *_≤ *m *≤ *m*_*i *_+ *rm*_*0 *_and *r*_*i *_- *rr*_*0 *_≤ *r *≤ *r*_*i *_+ *rr*_*0*_], the RANSAC window for row *i*.

Then, for row *i *in *L*, mark all peaks in sample S_*j *_in *RW*_*i *_as candidate alignments.

**Step 2**: Build a scatter plot representation of all candidate alignments, and apply the RANSAC algorithm to build a candidate model for alignment. This model represents a list of matching retention times.

**Step 3**: Apply the locally-weighted scatterplot smoothing (LOESS) method for regression [21] on all points in the model obtained with RANSAC.

**Step 4**: Using this regression model, for each row *i *in *L*, predict the correction for the retention time shift to locate the new center (*m*_*i*_*, r'*_*i*_) of the alignment window *AW*_*i*_. RANSAC alignment can correct the retention time deviation by centering the position of the *AW *to the correct position in the new sample.

Thus, the alignment window AW_i _= [(*m, r*) | *m*_*i *_- *rm*_*0 *_≤ *m *≤ *m*_*i *_+ *rm*_*0 *_and *r'*_*i *_- *ar*_*0 *_≤ *r *≤ *r'*_*i *_+ *ar*_*0*_]

**Step 5**: For each row i in L, apply the Join algorithm for alignment using the alignment window AW_i_.

**Figure 5:**
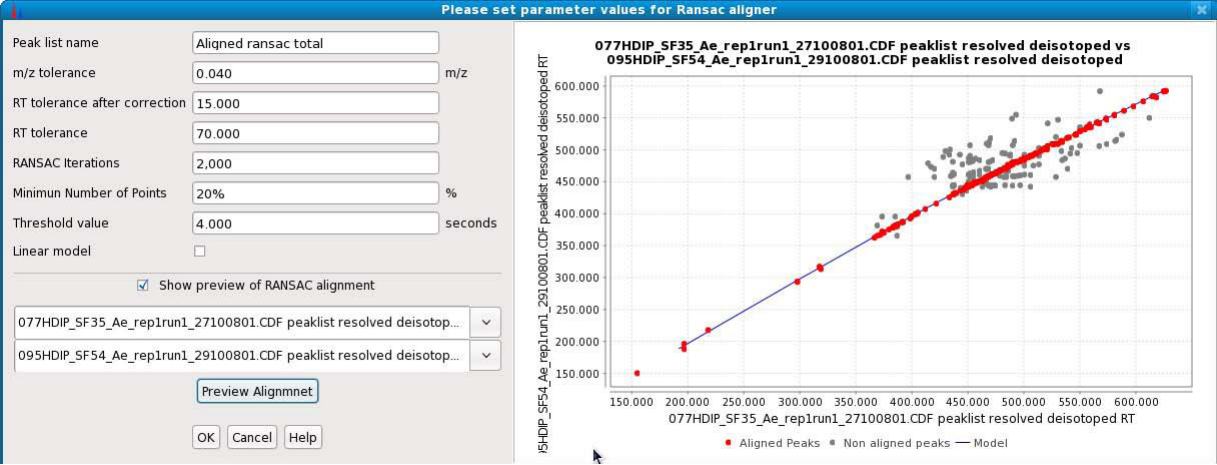
**RANSAC aligner**. Dialog shows preview of RANSAC alignment of two peak lists using the given parameters. Each possible candidate alignment (peak pair) within a defined m/z and retention time tolerance is shown as a dot. A model is fitted to the data (blue line) and red dots indicate those fitting to the model and therefore selected for the final alignment.

Figure 5 shows a preview of the RANSAC alignment in MZmine 2. Each dot represents a candidate alignment of two peaks. Red dots represent those candidate alignments that were fitted to the best model (blue line).

### RANSAC aligner performance

Two types of errors can be introduced during the alignment process [11]. Either two non-related peaks could be matched, or the matching of two related peaks could be omitted. A variable called **"precision" **represents the proportion of true alignments out of all alignments found by the algorithm. The proportion of peaks that are correctly aligned by the algorithm out of all true alignments inside the dataset is called "**recall**". These two variables together represent the quality of the alignment. To test whether the newly introduced RANSAC algorithm performs better than the Join alignment, the results of two different approaches were compared.

**Figure 6:**
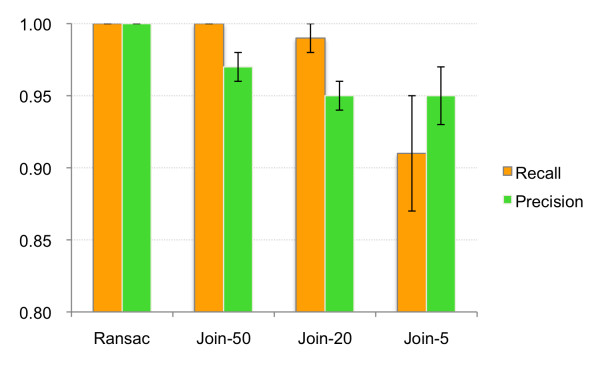
**Performance comparison of RANSAC aligner and Join aligner for 12 synthetic datasets**. For each dataset, peak lists were aligned using the RANSAC aligner and the Join aligner with three different retention time tolerance thresholds (50 s, 20 s, and 5 s). Plot shows the average recall and precision values for all datasets. Error bars indicate standard deviations.

First, 12 synthetic datasets were created using samples from 12 different lipidomic studies. A single sample from each study was used as a seed to create a synthetic set of 20 samples. These 20 samples contained identical information (peaks), but a random non-linear deviation in the retention time was introduced into each one. The MZmine 2 projects of all 12 datasets are available on-line (see Dataset download). Each dataset was aligned using the RANSAC aligner and Join aligner with three different retention time tolerance thresholds (50 s, 20 s, and 5 s). Parameters used for alignment are specified in Table 1. Run times of the RANSAC aligner were measured and are reported in Table 2. Precision and recall values were calculated and the average results are shown in Figure 6 (numerical results are available in Additional file 1). Only the use of the RANSAC algorithm achieved 100% in both precision and recall performance on these synthetic data sets.

**Table 1:** Parameter values used for aligning the 12 synthetic data sets and the real proteomic (P1 and P2) and metabolomic (M1 and M2) data sets using the RANSAC and Join aligners.

Parameter	12 synthetic data sets	Proteomics data		Metabolomics data	
		
		Data set P1	Data set P2	Data set M1	Data set M2
m/z tolerance	0.05 m/z	1.5 m/z	1.5 m/z	0.03 m/z	0.025 m/z

RT tolerance after correction	0:25	02:30	2:30	00:50	00:30

RT tolerance	0:50	03:30	05:00	00:30	00:30

RANSAC iterations	5000	50000	50000	15000	15000

Minimum number of points	20%	2.00%	0.10%	20.00%	20.00%

Threshold value	4 seconds	4 seconds	15 seconds	4 seconds	4 seconds

Non-linear model	yes	yes	no	yes	yes

**Table 2:** Run times of the RANSAC aligner for aligning the 12 synthetic data sets and the real proteomic (P1 and P2) and metabolomic (M1 and M2) data sets.

Data set		Run time (min)			
		
		Run 1	Run 2	Run 3	Average
Synthetic data set 1		0.17	0.15	0.16	0.16

Synthetic data set 2		0.32	0.31	0.31	0.31

Synthetic data set 3		0.44	0.41	0.42	0.42

Synthetic data set 4		0.46	0.45	0.45	0.45

Synthetic data set 5		0.62	0.67	0.74	0.68

Synthetic data set 6		0.39	0.38	0.39	0.39

Synthetic data set 7		0.54	0.54	0.55	0.55

Synthetic data set 8		0.25	0.26	0.25	0.25

Synthetic data set 9		0.79	0.80	0.84	0.81

Synthetic data set 10		0.73	0.73	0.72	0.73

Synthetic data set 11		5.24	4.11	5.17	4.84

Synthetic data set 12		7.79	7.78	7.64	7.74

M1		62.27	58.08	63.56	61.30

M2		147.64	163.62	146.79	152.69

P1	000	4.95	6.49	7.72	6.39
	
	020	0.50	0.50	0.57	0.52
	
	040	0.76	0.70	0.65	0.70
	
	060	1.11	1.06	1.14	1.10
	
	080	0.61	0.57	0.67	0.62
	
	100	0.46	0.48	0.51	0.48

P2	000	22.47	22.94	21.12	22.18
	
	020	1.35	1.31	1.18	1.28
	
	040	0.65	0.73	0.71	0.70
	
	080	0.31	0.36	0.39	0.35
	
	100	0.47	0.43	0.49	0.46

Run times were obtained on an AMD Opteron 1.8 GHz dual-core system with 10 GB RAM, running Linux.

Our second approach for the comparison was to use the real proteomic (P1 and P2) and metabolomic (M1 and M2) datasets introduced by Lange *et al. *[11], together with their tables of "ground truth" alignments and an evaluation script for calculating the alignment precision and recall values. We applied the MZmine 2 Join and RANSAC aligners to align all the datasets with the parameters specified in Table 1. Run times of the RANSAC aligner are reported in Table 2. Precision and recall values were calculated using the provided evaluation script and compared to already published results in Table 3. We used the latest available evaluation results published at http://msbi.ipb-halle.de/msbi/caap at the time of writing. Compared to the Join aligner, the RANSAC aligner provided better results in 11 of 13 alignments, with worse results obtained in only a single case (P2 dataset fraction 00). We assume that the high number of features in this fraction (over 6800 rows after alignment) made it somewhat difficult for the RANSAC algorithm to build a suitable model. Notably, in all fractions of dataset P1, the RANSAC aligner provided the best results among all the tested algorithms. Complete datasets P1, P2, M1, and M2, as well as all alignment results, are available online (see Dataset download).

**Table 3:** Performance comparison of MZmine 2 alignment methods (right side of the table) to previously published results (left side of the table) obtained using several different software packages [11].

	Results published by Lange et al. (2008), avaiable at the time of writing at http://msbi.ipb-halle.de/msbi/caap							MZmine 2 results	
			
	msInspect	MZmine (version 0.6)	OpenMS	SpecArray	XAlign	XCMS			
						
						without RT correction	With correction	Join aligner	RANSAC aligner
***Proteomics data set P1***									

**fraction 00**									

Recall	0.52	0.81	**0.86**	0.61	0.82	0.72	0.62	0.80	**0.86**

Precision	0.38	0.81	**0.86**	0.61	0.82	0.54	0.58	0.80	**0.86**

**fraction 20**									

Recall	0.56	0.90	**0.92**	0.62	0.85	0.88	0.81	0.90	**0.93**

Precision	0.45	0.90	**0.92**	0.62	0.85	0.84	0.80	0.90	**0.93**

**fraction 40**									

Recall	0.63	0.90	**0.94**	0.75	0.87	0.92	0.81	0.87	**0.94**

Precision	0.48	0.90	**0.94**	0.75	0.87	0.85	0.80	0.87	**0.94**

**fraction 60**									

Recall	0.73	0.84	**0.96**	0.71	0.87	0.91	0.78	0.89	**0.97**

Precision	0.54	0.84	**0.96**	0.71	0.87	0.80	0.75	0.89	**0.97**

**fraction 80**									

Recall	0.70	0.94	**0.96**	0.74	0.90	0.94	0.89	0.94	**0.97**

Precision	0.57	0.94	**0.96**	0.74	0.90	0.88	0.88	0.94	**0.97**

**fraction 100**									

Recall	0.82	0.94	0.94	0.77	**0.96**	0.95	**0.96**	0.95	**0.96**

Precision	0.56	0.94	0.94	0.77	**0.96**	0.89	**0.96**	0.95	**0.96**

***Proteomics data set P2***									

**fraction 00**									

Recall	0.23	0.62	**0.77**	0.07	0.65	0.70	0.58	0.63	0.56

Precision	0.07	0.49	**0.65**	0.05	0.49	0.31	0.44	0.53	0.49

**fraction 20**									

Recall	0.67	0.87	**0.92**	0.57	0.84	0.89	0.86	0.81	**0.93**

Precision	0.24	0.71	**0.77**	0.42	0.70	0.55	0.66	0.69	**0.78**

**fraction 40**									

Recall	0.44	0.79	0.76	0.60	0.71	0.72	0.72	0.74	**0.78**

Precision	0.26	0.76	0.74	0.41	0.69	0.56	0.69	0.73	**0.77**

**fraction 80**									

Recall	0.73	0.60	**0.80**	0.65	0.58	0.64	0.49	0.61	0.61

Precision	0.34	0.56	**0.70**	0.44	0.56	0.50	0.45	0.58	0.61

**fraction 100**									

Recall	0.82	0.80	0.90	0.63	0.85	**0.95**	0.85	0.85	0.88

Precision	0.39	0.64	**0.75**	0.44	0.69	0.65	0.69	0.71	**0.75**

***Metabolomics data sets***									

**M1**									

Recall	0.27	0.92	0.87	-	0.88	**0.98**	0.94	0.90	0.91

Precision	0.46	**0.73**	0.69	-	0.70	0.60	0.70	**0.74**	**0.74**

**M2**									

Recall	0.23	**0.98**	0.93	-	0.93	0.97	**0.98**	**0.98**	**0.98**

Precision	0.47	**0.84**	0.79	-	0.79	0.58	0.78	0.83	0.83

## Conclusions

The development of MZmine 2 was motivated by the need for a flexible and modular software platform that would allow the bioinformatic and analytical community to contribute new methods for specific stages of MS-based data processing. Great emphasis was placed on achieving the three main goals of a flexible, extendable, and modular design; user-friendly graphic interface; and good support for high-resolution MS data. The authors of this manuscript work in the field of metabolomics utilizing an LC-MS analytical platform, and therefore the currently developed modules were tested mainly on LC-MS data. The flexibility of MZmine 2, however, allows for easy expansion to other dataset types such as gas chromatography-MS, as well as interoperation with popular proteomics search engines such as MASCOT.

Several other software packages have been introduced for LC-MS based data processing, such as XCMS^2 ^[22], Trans Proteomic Pipeline [23], Trequips [24], OpenMS-TOPP [25], and ProteoWizard [26]. None of these tools, however, share the same goals with MZmine 2, most of them being command-line oriented with fixed feature sets, aiming specifically for either proteomic or metabolomic research. Rather then a single piece of software, the developmental aim of MZmine 2 is to create a universal platform through which researchers can contribute individual processing modules and implement and share novel ideas, spanning over multiple research fields and analytical methods.

MZmine 2 is available for download at the project WWW site, together with a printable manual, an animated tutorial, a module development tutorial, and further relevant project information such as a source code repository and developers' mailing list. The current version of the framework is already suitable for processing large batches of data, both for targeted and/or non-targeted analyses, and has been applied in metabolomic research [13,27].

### Dataset download

The data associated with this manuscript may be downloaded from ProteomeCommons.org Tranche using the following hash:

The hash may be used to validate the files were published as part of this manuscript's dataset, and to check that the data have not changed since publication.

## Availability and requirements

• **Project name**: MZmine 2

• **Project home page**: http://mzmine.sourceforge.net

• **Operating system(s)**: Platform independent

• **Programming language**: Java

• **Other requirements**: Java Runtime Environment (JRE) 1.6, Java3D

• **License**: GNU GPL

## Abbreviations

AW: Alignment window; LC-MS: Liquid chromatography-mass spectrometry; MS: Mass spectrometry; RANSAC: Random sample consensus; RW: RANSAC window

## Authors' contributions

TP designed the data model and overall architecture of the MZmine 2 framework and implemented most of the raw data visualization and peak identification modules. SC implemented the project serialization and RANSAC aligner. AVB implemented the peak detection module with previews, scatter plot and histogram visualizers, and isotope pattern support, and contributed to the online database search module development. MO participated in software testing and provided feedback on the framework design. All authors read and approved the final manuscript.

## Supplementary Material

Additional file 1 **Numerical values for Figure **6. Precision and recall values of RANSAC and Join aligner results for 12 synthetic data sets. Click here for file
